# A sample design for globally consistent biomass estimation using lidar data from the Geoscience Laser Altimeter System (GLAS)

**DOI:** 10.1186/1750-0680-7-10

**Published:** 2012-10-31

**Authors:** Sean P Healey, Paul L Patterson, Sassan Saatchi, Michael A Lefsky, Andrew J Lister, Elizabeth A Freeman

**Affiliations:** 1 US Forest Service, Rocky Mountain Research Station, Fort Collins, CO, 80526, USA; 2 NASA Jet Propulsion Laboratory, Pasadena, CA, USA; 3 Colorado State University, Colorado, CO, USA; 4 US Forest Service, Northern Research Station, Newtown Square, PA, 19073, USA

**Keywords:** Biomass, Forest monitoring, Remote sensing, Lidar

## Abstract

**Background:**

Lidar height data collected by the Geosciences Laser Altimeter System (GLAS) from 2002 to 2008 has the potential to form the basis of a globally consistent sample-based inventory of forest biomass. GLAS lidar return data were collected globally in spatially discrete full waveform “shots,” which have been shown to be strongly correlated with aboveground forest biomass. Relationships observed at spatially coincident field plots may be used to model biomass at all GLAS shots, and well-established methods of model-based inference may then be used to estimate biomass and variance for specific spatial domains. However, the spatial pattern of GLAS acquisition is neither random across the surface of the earth nor is it identifiable with any particular systematic design. Undefined sample properties therefore hinder the use of GLAS in global forest sampling.

**Results:**

We propose a method of identifying a subset of the GLAS data which can justifiably be treated as a simple random sample in model-based biomass estimation. The relatively uniform spatial distribution and locally arbitrary positioning of the resulting sample is similar to the design used by the US national forest inventory (NFI). We demonstrated model-based estimation using a sample of GLAS data in the US state of California, where our estimate of biomass (211 Mg/hectare) was within the 1.4% standard error of the design-based estimate supplied by the US NFI. The standard error of the GLAS-based estimate was significantly higher than the NFI estimate, although the cost of the GLAS estimate (excluding costs for the satellite itself) was almost nothing, compared to at least US$ 10.5 million for the NFI estimate.

**Conclusions:**

Global application of model-based estimation using GLAS, while demanding significant consolidation of training data, would improve inter-comparability of international biomass estimates by imposing consistent methods and a globally coherent sample frame. The methods presented here constitute a globally extensible approach for generating a simple random sample from the global GLAS dataset, enabling its use in forest inventory activities.

## Background

Methods are needed to monitor the magnitude and spatial distribution of global forest carbon storage, an important component of the global carbon cycle. Initiatives such as REDD (United Nations Collaborative Programmed on Reducing Emissions from Deforestation and Degradation in Developing Countries) depend upon accurate, precise, and consistent national-level reporting of forest carbon storage. Traditionally, estimates of carbon storage in the context of international monitoring have come from field-based inventories [1]. In such inventories, well-developed principles of sample design support straightforward derivation of estimates and uncertainties. However, many countries do not have national forest inventories, and among those that do, important differences in methods and definitions can exist.

Satellite-based forest monitoring may offer observations which are more consistent across space and time, and potential contributions of remotely sensed estimation of carbon stored in biomass are widely recognized [2,3]. However, barriers to broad acceptance of remotely sensed biomass estimates exist. Widely available satellite data, particularly from optical sensors such as Landsat and MODIS, may be relatively insensitive to different levels of biomass under closed forest canopies (e.g. [4,5]). More importantly, while credible efforts have been made to empirically propagate errors through the process of summing pixel-level biomass predictions at the national level (e.g. [6]), acceptance of such approaches lags behind more formal estimation methods.

Several efforts to move beyond these limitations have centered around the use of lidar (light detection and ranging), not in wall-to-wall mapping (which can be relatively expensive) but as a vehicle for forest sampling [7]. Lidar instruments measure characteristics of laser pulses as they return off of objects at different heights above the earth’s surface. The actively generated signals used by lidar typically penetrate deeper into the forest canopy than the passive signals used by optical sensors, and strong relationships are often found between lidar return data and forest structure parameters such as biomass and volume [8,9].

While local- to regional-scale lidar monitoring missions are typically flown with instruments mounted on fixed-wing aircraft, globally consistent monitoring may be best achieved with spaceborne lidar. To date, the only widely available source of spaceborne lidar has been the GLAS (Geosciences Laser Altimeter System) instrument on NASA’s ICESat (Ice, Cloud, and land Elevation) satellite, which gathered data from 2002 to 2008. GLAS’ “full waveform” measurements are based upon time variation in the intensities of returned laser pulses, which resolve elliptical areas approximately 65 meters in diameter. GLAS measurements (“shots”) have been shown to be strongly correlated with biomass [10], and earlier problems with data quality on steeper slopes have been addressed to the point where such measurements can now be used in vegetation monitoring [11].

**Figure 1:**
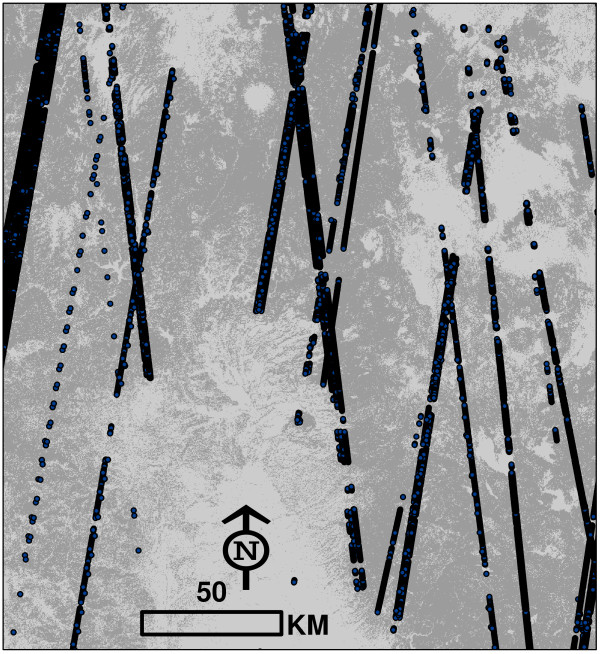
**Tracks of available ICESat/GLAS measurements in northern California.** Sample density varies arbitrarily across the state. Darker areas represent forested land cover classes [15].

The primary obstacle to widespread use of GLAS is its irregular acquisition pattern over forests. Points were acquired approximately 172 meters apart along the satellite’s flight path, which included both ascending and descending tracks on an orbit with a 94° declination [12]. In aggregate, data points from the GLAS mission exhibit a spatial pattern that is not clearly identifiable with any particular sample design (Figure 1). In almost all airborne lidar campaigns, acquisition follows a pre-determined sample framework (e.g. random or stratified; [13]), which informs the estimation process. In one of the only published efforts to utilize GLAS as a biomass sampling tool, Nelson et al. [14] tested a variance estimator which was design-unbiased for simple random sampling (SRS), and compared results to those obtained by estimators used in systematic sampling. However, it is debatable whether the spatial allocation of satellite tracks depicted in Figure 1 can appropriately be labeled either random or systematic. There are clearly areas of the map which are sampled more intensively than other, and an inability to explicitly define GLAS sample properties is an important barrier to use of the sensor in global biomass inventories.

In this paper, we propose a method for identifying a subset of GLAS shots which can be treated as a simple random sample. We then demonstrate the use of such a sample over the U.S. state of California with a model-based estimator similar to that used by Stähl et al. [16]. Model-based estimation, described below, allows us to predict, instead of measure, biomass at each sample point using relationships derived from a separate set of co-located ground and lidar measurements. Variance estimators used in this process take into account the uncertainty associated with the models used.

The sample design we describe is similar to that used by the U.S. national forest inventory (NFI), the Forest Inventory and Analysis program (FIA) administered by the U.S. Forest Service. Prior to a move toward a national sampling framework in the late 1990s, FIA plots were distributed and measured in slightly different ways in different regions of the country. The move to a nationally coherent sampling frame was accomplished by superimposing a hexagonal grid over the entire country, with the area of each grid cell equal to the nominal area represented by each FIA sample [17]. In cells where one existing plot fell, that plot was kept. In those with more than one plot, only one was selected at random for retention. In those with no existing plots, a plot was established in a random location.

Establishment of this semi-systematic, equal-area sample frame, therefore, allowed FIA to accommodate existing measurement locations while drawing a sample which was spatially balanced across the country and yet was random with respect to forest conditions [18]. The sample design we propose for GLAS follows a similar approach. One and only one GLAS shot is retained in each cell of an equal-area (but not equal-shape) tessellation of the area labeled as “forest” in a global land cover map. This tessellation is created following a fractal-based approach, using simple geometric rules to create equal-area clusters [19]. Since retroactively “adding” GLAS measurements (the last of which were collected in 2008) is not possible, tessellation cell size (and, inversely, sample number) is limited by the constraint that each equal-area cell must contain at least one GLAS shot.

Given the elimination of all GLAS shots except one in every tessellation cell under this approach, it is of practical interest to know the precision of resulting biomass estimates. The precision (i.e. standard error) of model-based estimates of biomass in California using the GLAS sample will be compared to design-based estimates derived from FIA’s sample of more than 5500 field measurements in the state.

## Results

**Figure 2:**
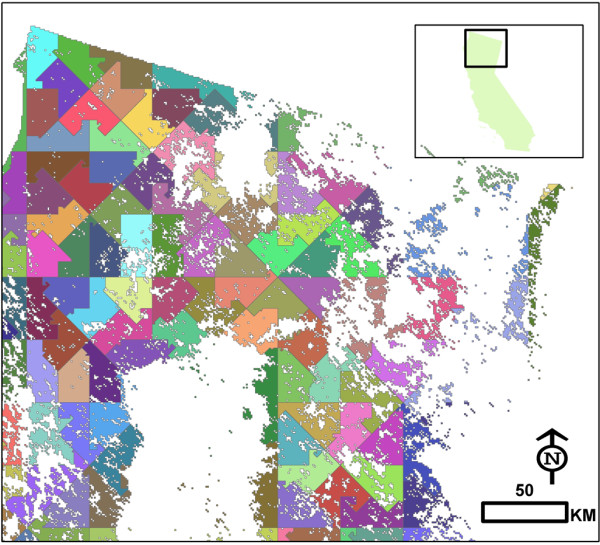
**Equal-area segments of the number line passing through each pixel center.** Shown is the segmentation where each segment represented 48,000 hectares, the smallest possible sample unit (and highest possible sample number) if each segment is to contain at least one GLAS shot. One randomly selected GLAS shot from each segment is included in the S1 sample (Figure 3).

**Figure 3:**
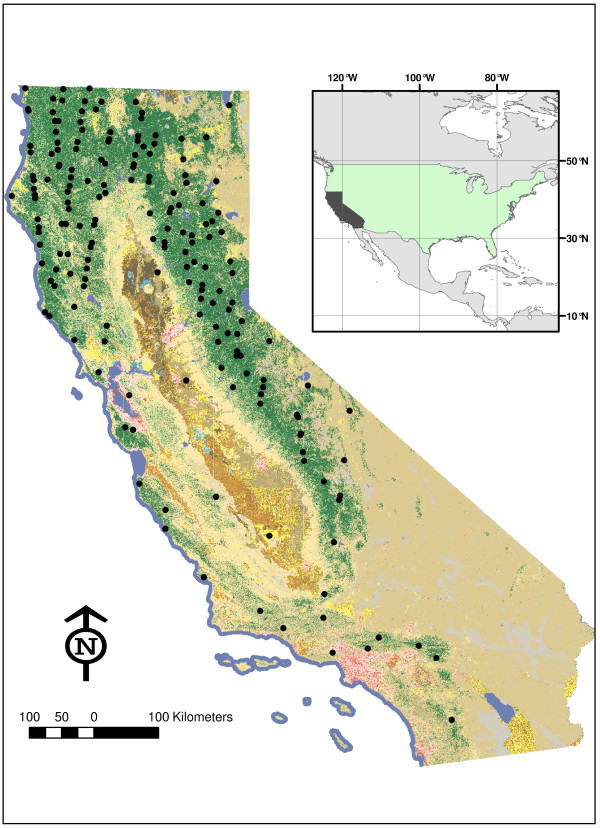
**The 182 GLAS shots selected for inclusion in the S1 sample of California forests.** This sample has properties similar to the sample used in the US NFI and is treated here as a simple random sample. A National Land Cover Database [15] cover map is shown for context.

In the sample design we propose, pixels in a map of the (forested) domain of interest are ordered along a number line according to a space-filling curve following simple geometric patterns. The goal is to generate what amounts to a tessellation of the study area into equal-area sample regions by dividing the pixel number line into equal-length segments, each of which contains at least one pixel which corresponds to a GLAS Lorey’s height measurement. Tests in California involving all possible segment lengths and potential segmentation starting points revealed that the smallest segment length which met the “1 GLAS shot per segment” rule was 9054 230-m pixels. Mapping these segments produced the pattern shown in Figure 2. There were 182 total tessellation cells, or one per 48,000 ha. While the minimum number of GLAS shots in a single cell was one, the average was 560, from which a single shot was chosen at random. These randomly selected shots, constituting the S1 sample, are displayed in Figure 3. The average distance between each point in the S1 population and its closest neighbor is 19.6 km (median = 13.5 km). The minimum overall distance (i.e. closest pair of neighbors) is 2.4 km.

**Figure 4:**
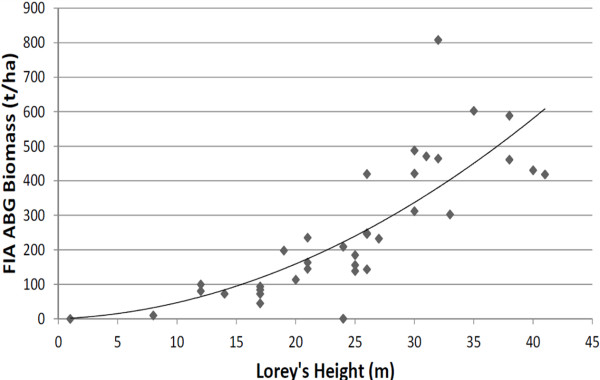
**The relationship FIA-measured aboveground tree biomass and GLAS Lorey’s height in California.** The line is described by: y = 0.3717 *x*^2^.

There were 35 co-located GLAS/FIA plots available for use in determining the relationship between Lorey’s height and biomass (i.e. the S2 sample; Figure 4). The most parsimonious applicable model for this relationship was considered to be a model with a single quadratic term and no intercept (biomass = 0.3717 (Lorey’s height)^2^). A no-intercept model was used because of our assumption that forested plots with no biomass should return no Lorey’s height. Significance tests indicated negligible gain of including a linear term in the model.

The *R*^2^ value the quadratic-only model was 0.87, although this figure should be viewed with the understanding that the *R*^2^ for a no-intercept model is calculated differently and represents a different aspect of correlation than *R*^2^ calculated for models which include an intercept term. To compare the two models, one can use a conditional *R*^2^, namely $R^{2}=\left[1-\sum {\left(Y-{\hat{Y}}_{Full\;model}\right)}^{2}/\sum {\left(Y-{\hat{Y}}_{Reduced\;model}\right)}^{2}\right]$, where the full model is the intercept model and reduced model is the no-intercept model. The value of this *R*^2^ is 0.001 in our case. Thus, for the sake of comparison with relationships observed by others between GLAS and aboveground tree biomass, the fit of the model was quite similar to that of a quadratic-plus-intercept model, for which the r^2^ value was 0.64.

It should be noted that seven values (less than 4%) of the S1 sample exceeded the largest value in the model-building S2 dataset shown in Figure 4 (specifically, these were values of: 45, 46, 48, 50, 52, 54, and 60 meters). Ideally, the model-building dataset should span the entire range of the values to be modeled. However, given the small percentage of Lorey’s heights in S1 not represented in S2, we assume that the model is valid for the entire population. We likewise assume no spatial autocorrelation among S1 samples.

**Figure 5:**
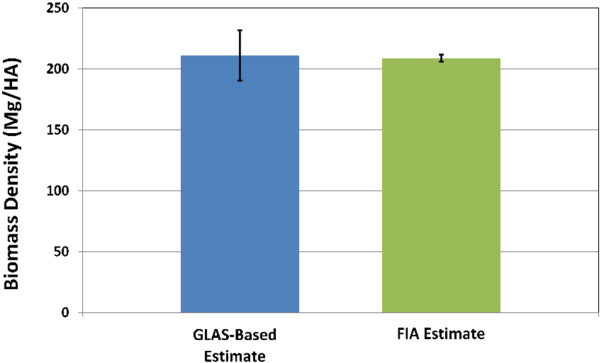
**Comparison between the FIA carbon density estimate for California’s forests and the estimate made here using GLAS and model-based estimation.** The estimates are nearly identical, although FIA’s estimate has significantly less uncertainty (bars indicate standard error).

Our GLAS-based estimate of biomass density in California’s forests was 211.11 Mg/ha, which was within standard error bounds (±2.88) of the FIA estimate of 208.95 Mg/ha [20]. The FIA estimate was derived through a 10-year ground sample of 5261 forested plots. The standard error of the GLAS-based estimate was 20.70 Mg/ha (Figure 5). The sample design portion of the variance estimation, the first summand of Equation 4, was 241.00. Since there is no intercept ${\hat{V}}_{S2}\left(\hat{\beta }\right)$ is equal to $\mathrm{MSE}/\underset{S2}{\sum }\;{\left(X_{i}^{2}\right)}^{2}$, which was equal to 0.0005776397. The product of ${\hat{V}}_{S2}\left(\hat{\beta }\right)$ and ${\left(\bar{x^{2}}\right)}^{2}={\left(\underset{S1}{\sum }\;X_{i}^{2}/n\right)}^{2}$ was 186.31; this is the value of the second summand in Equation 4 and is the contribution of the uncertainty in the model predictions to the estimated variance. The modeling variance is approximately 0.77 times the variance contributed by the sampling process.

## Discussion

Model-based estimation using the sample design we describe provides a transparent method for estimating biomass for particular spatial domains. This sample design, in which one arbitrarily located sample point is drawn from equal-area sample units distributed across the landscape, is similar to that used by FIA, and our estimate of biomass density in the state of California closely matched FIA’s design-based estimate. The standard error of our estimate (approximately 9.8% of the estimate) was substantially larger than that of the FIA estimate (1.4%) and that derived through model-based estimation by Andersen et al. [21] using specifically acquired airborne lidar data (2011; 8%). However, the cost of the FIA estimate was approximately $10.5 million (using a commonly used valuation of $2000 per plot), and the lidar acquisition alone in Anderson et al.’s much smaller study area cost $60,000. While NASA’s investment in the GLAS mission was considerable, future use of GLAS data in the process described here represents an almost no-cost option for providing consistent, moderate-precision biomass estimates across the globe.

A primary advantage of the model-based inference used here is the capacity to apply models developed in areas of rich inventory data to GLAS shots informing estimates in ecologically similar areas where field data are sparse. For example, Nelson et al. [14] used relationships observed in a limited area of co-located biomass/GLAS observations to estimate biomass for the entire Canadian province of Quebec, following a modified model-based approach. However, the validity of inference in model-based approaches depends upon how well the stipulated models accord with the population of interest [22]. The question of how well the model “applies” to the population of interest is a critical consideration in the application of our approach, whether the model was developed *in situ* or from a spatially remote but perhaps ecologically similar area. Since our model was created from an arbitrary subset of FIA’s presumably unbiased ground sample, there is a compelling argument that the model is appropriate for the forests of California.

The degree to which this model may apply beyond California remains an open question. Saatchi et al. [6] noted regional differences in the relationship between biomass and GLAS Lorey’s height in their pan-tropical study. Data collected to support biomass estimation using the global GLAS dataset would at least have to span major ecological systems. The need for broadly collected ground measurements in the composition of our S2 sample highlights the fact that there will always be demand for up-to-date ground data. Model-based estimation may spatially extend the value of available field data, but models must ultimately be grounded in actual observations that are relevant to the domain of interest.

The consolidation of ground data needed to support a global GLAS-based biomass inventory would require significant international cooperation and, as illustrated by our results, would likely not improve the precision of biomass estimates available in countries with established National Forest Inventories (NFIs). NFIs typically rely upon a denser sample than is available from GLAS, and do not have to account for model variance, which in our example made up approximately 44% of the total variance.

However, a GLAS-based biomass inventory would represent an internationally coherent basis for comparison among countries, especially those without established NFIs. Even moderate-precision biomass estimates would represent an improvement in many countries [2], and consistent sample design and estimation methods would remove an important source of uncertainty in international monitoring. The ICESat-2 mission, due in 2016, may provide an opportunity to update any GLAS-based biomass monitoring system. Although the scanning sensor on the ICESat-2 platform will provide continuous sample lines instead of discrete waveform returns, similar acquisition patterns from airborne lidar instruments have been discretized and used in model-based estimation approaches [16,21].

An important variable not considered in this paper is how the area of forest is determined. As stated earlier, the domain of our estimation was the area in California mapped as “forest” by the MOD12Q1 global land cover product. However, significant disagreements can exist among land cover maps [23], due both to varying definitions and alternative mapping methods. Use of different maps may result in different S1 sample sizes, varying biomass density estimates, and different overall carbon estimates as density values are multiplied by mapped forest totals. Bearing in mind that the forest cover map used in this methodology functions as a proxy for the true distribution of forest, it is important to choose a map which best serves analytical needs. For international inventory purposes, it is reasonable to use a globally consistent product such as MOD12Q1.

In view of international efforts to increase or preserve forest carbon storage, the global GLAS height dataset presents an opportunity to establish how forest biomass was distributed internationally in 2005 (the mid-point of the GLAS mission). GLAS data were acquired in spatial patterns difficult to associate with either a systematic or random process. The sample design presented in this paper allows identification of a subset of GLAS data which may be used as a simple random sample to estimate biomass, perhaps globally, with consistent measures of uncertainty under a model-based estimation framework.

## Conclusions

● The methods presented here constitute a globally extensible approach for generating a simple random sample from the global GLAS dataset. The properties of the sample collected by GLAS have hitherto not been strictly identifiable with any particular design.

● Model-based estimation, following Stähl et al. (2011), based upon GLAS data in the state of California produced an estimate of biomass density (biomass/hectare) almost identical to the estimate derived from the design-based NFI.

● Global application of model-based estimation using GLAS, while demanding significant consolidation of training data, would improve inter-comparability of international biomass estimates by imposing consistent methods and a globally coherent sample frame.

## Methods

### GLAS processing

GLAS shots acquired in the following collections were intersected with the global MOD12Q1(v004) MODIS land cover product, subset for the state of California: L3B, L3C, L3D, L3E, L3F, L3G, L3H, and L3I. Shots were kept if they fell over one of five forest classes (“evergreen needle leaf”, “evergreen broadleaf”, “deciduous needle lead”, “deciduous broadleaf”, and “mixed”). This area became the domain over which average biomass density (tones/hectare) was to be estimated.

Shots were filtered only on the basis of quality flags due in many cases to clouds or other atmospheric anomalies. Topographic correction was applied following Lefsky et al. [11]. Full-waveform signatures were processed to a crown-weighted height metric called “Lorey’s height” [24]. Lorey’s height, used recently in a global tropical biomass mapping project [6], was the GLAS derivative upon which subsequent modeling and estimation were based.

### A sample design for GLAS data

The choice of a particular statistical estimator does not necessarily imply any particular sample design [22]. The model-based approach to inference that we describe in the next section has been employed with airborne lidar data, often using sample designs which consider strips of lidar measurements as systematic cluster samples (e.g. [16,21]). However, as illustrated above, the irregular positioning of GLAS ground tracks poses difficulty in defining the terms under which the sample can be considered representative of the population. The primary contribution of this paper, which we describe in this section, is a means of identifying a subset of GLAS data which can be treated as a simple random sample in the estimation process.

Four steps were involved with this process:

1. Assign an ordinal number to each pixel in the forest map representing the domain of interest. The MODIS product referenced above was re-sampled from its native 1-kilometer resolution to 230 meters so that processing would occur at a scale closer to the field of view of the GLAS shots (approximately 70 meters). Re-sampling to 230 meters produced over 1.6 million pixels in the California study area, which, given subsequent operations, was near local computing limits. Next, a space-filling curve [19] was applied through the center point of each “forest” pixel to generate an ordered list of pixel locations. This fractal-based ordering process (described in detail in [19]) involved the generation of a self-similar line (Piano curve) that folded in upon itself as it occupied the set of pixel centers found on the landscape.

2. Align GLAS-based Lorey’s heights with spatially correspondent pixels on the ordinal number line. The GIS coverage of GLAS shots was spatially intersected with the ordered network of forested pixel centers in a combination of a GIS and Microsoft Access processes, and the Lorey’s heights were added to approximately 102,000 of the 1.6 million locations represented on the number line. In cases where multiple GLAS points fell within a single pixel, one was chosen at random as representative.

3. Divide the ordered number line into equal-length segments, such that there is at least one Lorey’s height measurement associated with each segment. A script was written using the open-source R statistical programming language [25], in which the ordered list of forested pixel centers (i.e., the number line) was iteratively broken into equal segments of varying length and tested for the condition of containing at least one pixel associated with a GLAS shot. This was accomplished by transforming the line into a matrix of *n* columns made up of equal contiguous line segments of lengths *l*, with the total length of the number line equal to *n l* plus a remainder, which was ignored. A matrix was considered a viable solution if each column contained at least one pixel center point with an associated GLAS measurement (see step 2).

Matrices representing different segment lengths were tested, starting with the shortest possible segment satisfying the requirement of ≥1 GLAS shot per segment (i.e. one half the length of the longest gap between GLAS shots on the number line) and working upward until a viable solution was found. Since the location of the first pixel represented in the number line was arbitrary, all possible segmentation starting points were tested for every tested segmentation length, “looping” the end of the number line to the beginning. R code for each of these operations has been uploaded to the Journal archive ( Additional File 1: “gap_finding_and_segment_sampling_R_code.pdf”).

4. For segments associated with more than one GLAS shot, choose one at random for the sample.

Similar to FIA’s sample design, this process assures a relatively uniform spatial distribution of plots but allows locally random positioning of measurements. Following FIA’s precedent [18], this sample is treated subsequently as a simple random sample.

### Model-based estimation

Model-based inference depends upon fundamentally different assumptions than the design-based methods used by most field-based inventories, including FIA’s (for detailed description of the difference between model- and design-based inference, see [22]). Unlike design-based estimation, model-based methods treat observations as realizations of a random process (model).

The model-based approach we follow is similar to that of Stähl et al. [16]. We make use of two samples; sample S1 is the “application sample” developed in the steps above, for which modeled Lorey’s heights are the only data available; and, sample S2, which is composed of co-located field and GLAS measurements which can be used to build and assess biomass models to be applied at all S1 plots. In this study, the S2 sample was not a subsample of S1; S2 was made up of the 35 single-condition, forested FIA ground plots in California which had plot centers falling within 120 meters of the center of a GLAS shot and which did not fall along condition boundaries, as determined by visual inspection of high-resolution National Land Cover Database maps [15]. Care was taken to avoid condition boundaries to minimize mismatch in the forest measured by the satellite and forest measured in the field.

The relation between Lorey’s height *x* and biomass *Y* was assumed to follow a linear regression model

(1) $$Y=\overset{p}{\underset{j=0}{\sum }}\beta_{j}x_{j}+\varepsilon ,with\;\varepsilon \sim N\left(0,\sigma^{2}\right)$$

The predicted value of biomass *Ŷ* was constructed using maximum likelihood estimates based on the *S*2 sample, denoted by the parameter estimates ${\hat{\beta }}_{j}$. The parameter estimates were constructed using linear model package, lm, in the R programming language [25]. By standard theory for linear models [26], *Ŷ* is an unbiased estimator of the expectation of *Y* and there is an unbiased estimator of the variance covariance matrix of the parameter estimates, and an unbiased estimate $\hat{V}\left(\hat{Y}\right)$ of *V*(*Ŷ*), the variance of *Ŷ*.

We assume a finite population model, where the population element is the land associated with each 230-meter pixel. The term “pixel” will be used to refer to the land associated with the pixel. It is of interest to estimate the population mean $N^{-1}\sum Y_{i}$, where *Y*_*i*_ is the biomass in Mg per hectare for the *i*^th^ pixel and *N* is the number of elements in the population. We proposed using the predicted biomass values of the *S*1 sample elements to estimate the population mean, in particular

(2) $$\bar{\hat{y}}=\frac{1}{n}\sum_{S1}{\hat{Y}}_{i}$$

where *Ŷ*_*i*_ is the predicted biomass value for the *i*^th^ element of *S*1 and *n* is the number of elements in *S*1. The estimator is an unbiased estimator of the population mean of the expected value of the *Ŷ*_*i*_ with respect to the sampling distribution; that is: $E\left(\bar{\hat{y}}\right)=N^{-1}\sum_{i}^{N}E_{S2}\left({\hat{Y}}_{i}\right)$. The bias in the estimate is $N^{-1}\sum_{i}^{N}e_{i}$, where *e*_*i*_ is the value of unknown error, with the expected value of this bias equal to zero.

In accounting for the sources of uncertainty we assumed the model form was correct; that is, there was no uncertainty due to incorrect specification of the model. Also we assumed the Lorey’s height was measured without error. When the uncertainty due to the sampling design of *S*1 and the uncertainty due to the sampling distribution of the predicted value *Ŷ* were taken into account, the variance of $\bar{\hat{y}}$ is

(3) $$\begin{array}{l} V\left(\bar{\hat{y}}\right)=V_{S1}\left(\bar{E_{s2}\left(\hat{Y}\right)}\right)\\ \;+\sum_{k=0}^{p}\sum_{l=0}^{p}C_{S2}\left({\hat{\beta }}_{k},{\hat{\beta }}_{l}\right)E_{S1}\left({\bar{x}}_{*k}{\bar{x}}_{*l}\right) \end{array}$$

where *E*_*S*1_ and *V*_*S*1_ are the expectation and variance with respect to the sample design of *S*1, $\bar{E_{S2}\left(\hat{Y}\right)}$ is the mean over the sample *S*1 of $E_{S2}\left({\hat{Y}}_{i}\right)=\sum_{j=0}^{p}\beta_{j}x_{\mathrm{ij}}$, *C*_*S*2_ is the covariance with respect to the sampling distribution of the parameter estimates of the linear model [26], and ${\bar{x}}_{*k}$ is the mean over *S*1 of the *k*^th^ component of the *x* vector [16]. By Ståhl et al. [16], an approximately unbiased estimator of $V\left(\bar{\hat{y}}\right)$ is given by

(4) $$\hat{V}\left(\bar{\hat{y}}\right)={\hat{V}}_{S1}\left(\bar{\hat{Y}}\right)+\sum_{k=0}^{p}\sum_{l=0}^{p}{\hat{C}}_{S2}\left({\hat{\beta }}_{k},{\hat{\beta }}_{l}\right){\bar{x}}_{*k}{\bar{x}}_{*l}$$

where $\hat{V}$ is the standard estimate with respect to simple random sampling of the variance of the mean. Given the form of the model used in this case $\left(Y=\beta x^{2}+\varepsilon ,\text{with}\;\varepsilon \sim N\left(0,\sigma^{2}\right)\right)$, the double sum $\sum_{k=0}^{p}\sum_{l=0}^{p}{\hat{C}}_{S2}\left({\hat{\beta }}_{k},{\hat{\beta }}_{l}\right){\bar{x}}_{*k}{\bar{x}}_{*l}$ collapses to ${\hat{V}}_{S2}\left(\hat{\beta }\right){\left(\bar{x^{2}}\right)}^{2}$ because there is only one term in the sum. R code for the model-building and estimation processes is given in Additional File 2 (1-modelBuilding_and_biomass_estimation_R_code.pdf).

## Competing interests

The authors declare that they have no competing interests

## Authors’ contributions

SPH conceived of the study and composed much of the manuscript. PLP performed and described the statistics and modeling components of the study. SSS contributed to study design, while MAL was responsible for all GLAS data processing. AJL adapted the space-filling curve technique to the GLAS data in California, and EAF conceived of and performed the process of finding the segmentation of the state which maximized the sample size. All authors read and approved the final manuscript.

## Supplementary Material

Additional File 1 **This R code was used in the process described under the heading “Divide the ordered number line into equal-length segments, such that there is at least one Lorey’s height measurement associated with each segment” in the Methods section.** Code is presented in the hope that it might be useful to those replicating this process. Click here for file

Additional File 2 **This R code was used to develop the Lorey’s height/biomass relationship drawn from the S2 sample.** Also included are calculations involved with model-based estimation of biomass. Click here for file
