# Supplementary material for: A sample design for globally consistent biomass estimation using lidar data from the Geoscience Laser Altimeter System (GLAS)
Source: Carbon Balance Manag. 2012 Oct 31;7:10. doi: 10.1186/1750-0680-7-10 (PMC3527180; doi:10.1186/1750-0680-7-10)
Supplement: Additional File 1 — This R code was used in the process described under the heading “Divide the ordered number line into equal-length segments, such that there is at least one Lorey’s height measurement associated with each segment” in the Methods section. Code is presented in the hope that it might be useful to those replicating this process. [file 1750-0680-7-10-S1.pdf]

```
file<-"Output.txt"

### GL  Gap Length (of langest Gap)
### GB  Gap Beginning (of longest Gap)

### SL  Segment Length
### SB  Segment Beginning


##################### Read Data ###################################

DATA<-read.csv("final_order_glas.csv",header=T)
head(DATA)


###### Find length and beginning location of largest Gap ##########

X<-DATA$LC
N<-length(X)

ONES<-DATA$g230_ID[DATA$LC==1]
N.ones<-length(ONES)

DIFF<-rep(0,N.ones)
DIFF[1]<-ONES[1]-1
DIFF[2:N.ones]<-ONES[2:N.ones]-ONES[1:(N.ones-1)]-1

STARTS<-rep(0,N.ones)
STARTS[1]<-1
STARTS[2:N.ones]<-ONES[1:(N.ones-1)]+1


#If there are multiple gaps of the largest gap length, take the first gap
GL<-max(DIFF)[1]
GB<-STARTS[DIFF==max(DIFF)][1]

GL
GB

#delete DATA to free up memory
rm(DATA)

#final numbers:
#GB<-632751
#GL<-15901

########### Find length and starting point of shortest segment #######
############## such that all segments contain at least one 1 #########

# this requires slightly different code depending on whether the longest
# gap contains an odd or even length

##############
### ODD GL ###
##############

# check if odd
if(!identical((GL/2),trunc(GL/2))){

        ####################

        SLmin<-GL/2 + 1.5 # Smallest segment to test - half the longest gap length
```

```
plus 1.5
        SLmax<-GL+1       # Largest segment to test  - longest gap length plus 1

        SLmin
        SLmax
        ####################
        #set up text file to track progress
        cat("STARTING LOOPS \n", file = file, append = FALSE)

        DONE<-FALSE

        #Loop through possible segment lengths (starting with GL/2+1.5 and going up
to GL+1)
        i<-0

        while(!DONE && (i < ((GL+1)/2)) ){

                i<-i+1

                SL<-((GL+1)/2)+i
                Nseg<-trunc(N/SL)
                print(paste("SL =",SL))
                cat("     testing SL =", SL, "\n", file = file, append = TRUE)

                #Loop through possible segment begining (starting at center of gap
and working out in both dirrections)
                j<-0
                while(!DONE && (j < (2*i))){
                        j<-j+1

                        SB<- GB +((GL+1)/2) - (i+1) + j

                        cat("          testing SB =", SB, "\n", file = file, append
= TRUE)

                        Xmat<-matrix(   X[c(SB:N,1:(SB-1))][1:(Nseg*SL)],
                                        Nseg,
                                        SL,
                                        byrow=TRUE)
                        M<-matrix(1,SL,1)

                        if(all(Xmat%*%M>0)){
                                print("DONE DONE DONE")
                                print(paste("GL =",GL))
                                print(paste("GB =",GB))
                                print(paste("SL =",SL))
                                print(paste("SB =",SB))
                                DONE<-TRUE
                        }
                        rm(Xmat)
                        rm(M)
                }
        }

        N-Nseg*SL

        cat("GB =", GB, "\n", file = file, append = TRUE)
        cat("GL =", GL, "\n", file = file, append = TRUE)
        cat("SB =", SB, "\n", file = file, append = TRUE)
        cat("SL =", SL, "\n", file = file, append = TRUE)

        cat("Leftover =", N-Nseg*SL, "\n", file = file, append = TRUE)
}
```

```
################################################################################

###############
### EVEN GL ###
###############
# check if longest gap is even
if(identical((GL/2),trunc(GL/2))){

        SLmin<-GL/2 + 1 # Smallest segment to test - half the longest gap length
plus 1
        SLmax<-GL+1     # Largest segment to test  - longest gap length plus 1


        SLmin
        SLmax
        #######################

        #set up text file to track progress
        cat("STARTING LOOPS \n", file = file, append = FALSE)

        DONE<-FALSE

        #Loop through possible segment lengths (starting with GL/2+1 and going up to
GL+1)
        i<-0
        while(!DONE && (i < ((GL/2)+1))){

                i<-i+1

                SL<-(GL/2)+i
                Nseg<-trunc(N/SL)
                print(paste("SL =",SL))

                #Loop through possible segment beginings (starting at center of gap
and working out in both dirrections)
                j<-0
                while(!DONE && (j < (2*i-1))){
                        j<-j+1

                        SB<- GB + (GL/2) - i + j
                        print(paste("     SB =",SB))

                        Xmat<-matrix(   X[c(SB:N,1:(SB-1))][1:(Nseg*SL)],
                                        Nseg,
                                        SL,
                                        byrow=TRUE)
                        M<-matrix(1,SL,1)

                        if(all(Xmat%*%M>0)){
                                print("DONE DONE DONE")
                                print(paste("GL =",GL))
                                print(paste("GB =",GB))
                                print(paste("SL =",SL))
                                print(paste("SB =",SB))
                                DONE<-TRUE
                        }
                        rm(Xmat)
                        rm(M)
                }
        }

        N-Nseg*SL
        cat("GB =", GB, "\n", file = file, append = TRUE)
```

```
        cat("GL =", GL, "\n", file = file, append = TRUE)
        cat("SB =", SB, "\n", file = file, append = TRUE)
        cat("SL =", SL, "\n", file = file, append = TRUE)

        cat("Leftover =", N-Nseg*SL, "\n", file = file, append = TRUE)
}


##########Looking at final results###############

# Final Numbers:
SL<-9010
SB<-641746

GB<-632751
GL<-15901

LEFTOVER<-2884

######## Read in DATA ############

DATA<-read.csv("final_order_glas.csv",header=T)
head(DATA)

X<-DATA$LC
N<-length(X)
Nseg<-trunc(N/SL)

################# randomly sample 1 LC=1 per segment ###################

# rearrange DATA so that row 1 is SB
DATA<-DATA[c(SB:N,1:(SB-1)),]

# add factored column to DATA that indicates which segment each row is in
# note: leftovers are in segment NA
DATA$SegNum<-NA
DATA$SegNum[1:(Nseg*SL)]<-rep(1:Nseg,each=SL)
DATA$SegNum<-factor(DATA$SegNum)

head(DATA)                      #look at first few rows
tail(DATA)                      #look at last few rows

#reorder data by ID code
DATA<-DATA[order(DATA$g230_ID),]

head(DATA)                      #look at first few rows
tail(DATA)                      #look at last few rows
DATA[(SB-10):(SB+10),]  #look at rows surrounding SB


#Note: DATA ID forced into character mode then back into numeric
#to avoid issues with sample() in segments with single LC=1
SAMPLE<-tapply(as.character(DATA$g230_ID[DATA$LC==1]),DATA$SegNum[DATA$LC==1],sample
,size=1)
SAMPLE<-as.numeric(SAMPLE)

DATA.SAMPLE<-DATA[DATA$g230_ID%in%SAMPLE,]

#Check that there is one and only one sample per segment (should be TRUE)
all(table(DATA.SAMPLE$LC,DATA.SAMPLE$SegNum)==1)

#Check that all segments are represented(should be TRUE)
all((1:Nseg)%in%DATA.SAMPLE$SegNum)
```

```
###write csv file of DATA with added segment column###
write.csv(DATA,file="DATA9010.csv",row.names=F)

###write csv of sampled rows###
write.csv(DATA.SAMPLE,file="DATA9010sample.csv",row.names=F)
```
