# Supplementary material for: A sample design for globally consistent biomass estimation using lidar data from the Geoscience Laser Altimeter System (GLAS)
Source: Carbon Balance Manag. 2012 Oct 31;7:10. doi: 10.1186/1750-0680-7-10 (PMC3527180; doi:10.1186/1750-0680-7-10)
Supplement: Additional File 2 — This R code was used to develop the Lorey’s height/biomass relationship drawn from the S2 sample. Also included are calculations involved with model-based estimation of biomass. [file 1750-0680-7-10-S2.pdf]

```
############ DATA INPUT AND CALCULATED VARIABLE CONSTRUCTION ####################

  ### S2 DATA - USED TO CONSTRUCT MODEL
s2.data <-
read.table("C:/1Ogden_cur/MeasErrorInModeledVars/GlassAnalysis/final_P2_toPaul.csv",
header=TRUE, sep = ",", dec = ".", skip = 0, row.names = NULL);

s1.data <-
read.table("C:/1Ogden_cur/MeasErrorInModeledVars/GlassAnalysis/glas_final_toPaul.csv
",
header=TRUE, sep = ",", dec = ".", skip = 0, row.names = NULL);


 ###  S1 VARIABLES ###
names(s1.data)[1]<-"loreysh";
loreysh.sq <- s1.data$loreysh**2;
s1.data <- cbind(s1.data,loreysh.sq);
rm(loreysh.sq);


 ###  S2 CALCULATED VARIABLES ###
names(s2.data)[2]<-"loreysh";
loreysh.sq <- s2.data$loreysh**2;
s2.data <- cbind(s2.data,loreysh.sq);
rm(loreysh.sq);
s2.data <- s2.data[order(s2.data$loreysh),]

############ END DATA INPUT

##########
#   MODEL EXPLORATION AND BUILDING
#################

 ############ ZERO MODEL
zero.rss<-sum(s2.data$loreysh.sq**2)

 ############  INTERCEPT ONLY MODEL

int.model <- lm(s2.data$bio_AG ~ 1);

summary(int.model)

int.rss<-sum(int.model$residuals**2)
int.mse<-int.rss/(length(int.model$residuals)-1)

 ############ QUADRATIC TERM NO-INTERCEPT MODEL

quad.model <- lm(s2.data$bio_AG ~ s2.data$loreysh.sq - 1);

summary(quad.model)

par(mfrow=c(2,2));
plot(quad.model);
par(mfrow=c(1,1))

quad.rss<-sum(quad.model$residuals**2)
quad.mse<-quad.rss/(length(quad.model$residuals)-1)


 ############ QUADRATIC TERM WITH INTERCEPT MODEL
```

```
quad.int.model <- lm(s2.data$bio_AG ~ s2.data$loreysh.sq);

summary(quad.int.model)

quad.int.model.rss<-sum(quad.int.model$residuals**2);
quad.int.model.mse<-sum(quad.int.model$residuals**2)/(length(quad.int.model$residual
s)-2)


######## RESIDUAL SUMS OF SQUARES AND CONDITIONAL R^2  ###########

zero.rss  # Y=0 model
int.rss # Y=a model
quad.rss  # Y=bx^2 model
quad.int.model.rss # Y= a + bx^2 model

#r^2 for Y= a + bx^2 model conditional on bx^2 model
1-quad.int.model.rss/quad.rss

##########
#   END
#   MODEL EXPLORATION AND BUILDING
#################

######### ESTIMATION  #####################

a.2<-quad.model$coefficient[1]

quad.Biomass.Mg.ha <-  mean( a.2*s1.data$loreysh.sq );
quad.SampVar <-  var( a.2*s1.data$loreysh.sq )/length(s1.data$loreysh.sq);
quad.ModelingVar <-  quad.mse * mean(s1.data$loreysh.sq)**2 / zero.rss

quad.Biomass.Mg.ha # Estimate of Biomas per Hectare
quad.SampVar  # Sample variance of estimate of Biomass per Hectare
sqrt(quad.SampVar) # Sample SE of estimate of Biomass per Hectare

quad.ModelingVar  # Estimate of component of variance due to model error
sqrt(quad.ModelingVar) # Estimate of standard deviation due to model error
quad.ModelingVar/quad.SampVar  # comparing size of sample variance and modeling
variance
 # SE for sampling error plus uncertainity of model projection
sqrt(quad.SampVar + quad.ModelingVar)
```
